# From Lineage Property to Individual Property: Dynamics of Land Management in the Villages of Nasso and Dindéresso, Hauts-Bassins, Burkina Faso

**DOI:** 10.12688/openresafrica.16420.1

**Published:** 2026-03-02

**Authors:** Abdoulaye Sawadogo

**Affiliations:** 1 Sociologie, Universite Joseph Ki-Zerbo, Ouagadougou, Centre Region, 09 BP 293 Ouagadougou 09, Burkina Faso

**Keywords:** land dynamics, lineage property, land ownership, land tenure, Burkina Faso

## Abstract

**Background:**

This article analyses the dynamics of relations between communities and land resources in a context of scarcity resulting from land speculation. Faced with this reality, the changes brought about by social dynamics in the land sector have ultimately led to the introduction of new forms of land ownership in the villages of Nasso and Dindéresso in Burkina Faso. This issue is a corollary of spatial expansion following urban sprawl due to population growth and speculation around peri-urban land. With increased interest in peri-urban land, traditional management norms are eroding, giving way to more modern management norms that offer more power to individuals.

**Methods:**

This research is based on a qualitative approach using data from documentary reviews, semi-structured interviews with community and administrative actors, and direct observation. This approach has made it possible to analyse land management through the prism of public policies and internal community dynamics. Property rights theory has been used to examine this issue.

The combination of methodological and theoretical approaches has yielded illustrative results.

**Results:**

These results reveal the nature and challenges of the fragmentation of land ownership rights within communities. To this end, it appears that land dynamics have had a significant impact on forms of intra-community land ownership, leading to the individualisation of land rights following the advent of land commodification.

**Conclusions:**

In conclusion, it appears that land is a cross-cutting issue that impacts all spheres of society. Hence the multiple and multifaceted challenges in terms of land governance and the consolidation of social cohesion.

## Introduction

Land governance involves internal social relations within local rural society, as well as relations between the State and its citizens. These relationships become less harmonious as land exploitation intensifies and land potential becomes scarce. This situation is even more concerning when the race for land is driven by the quest for land for agricultural and pastoral use, as well as housing needs resulting from population growth and urban sprawl. In a context where cities are expanding horizontally, the annexation of rural lands brings new actors into the land arena. Today, rural residents and urban actors with land interests in rural areas are increasingly clashing ( Lavigne-Delville, 2020).

Exclusively under customary authority in the past, land management has undergone several waves of mutation. In the Burkinabè context, new land management practices were introduced following colonization. This policy took shape as early as 1899 in the colonies of French West Africa ^1^ (AOF) with the introduction of a political, social, and economic management model for people and the various resources found within the conquered territory. Thus, Upper Volta, present-day Burkina Faso, saw its first written texts on the reorganization of land ownership with the adoption of a decree on July 24, 1906, which introduced the land registration system. This decree was repealed and replaced by the land decree of July 26, 1932, which followed the continuity of previous texts ( Ouattara, 2014). It should be noted that the advent of these texts did not reflect an absence of governance standards for natural resources, especially since there were no vacant lands, much less territories without norms. Following colonial policy, newly independent states continued with centralized legal provisions that contrasted with the social logics governing territoriality and natural resource governance. This reality immerses us in a legal dualism, characterized by the coexistence of modern land rights and customary land rights, with a pluralism of norms in land administration. Faced with this reality, land governance in the rural world, and even sometimes in urban areas, is centered on a system of dual governance, divided between two logics based on different principles: the modern conception and the traditional conception of land ( Karambiri, 2022).

In a context of legal dualism, an approach in terms of “ownership” according to Lavigne-Delville (2002) is fundamentally ill-suited, or at least a source of confusion; to better understand land tenure, the author suggests it is better to speak of “land appropriation,” a term that does not presuppose the types of rights involved. Consequently, a question captured by Kaboré (2020) examines how these policies, through their development and implementation processes, offer various categories of actors’ opportunities to overcome the socio-land problems induced by the intensification of land acquisitions by new actors. This issue raises questions that pace the daily lives of populations in the peripheral villages of the city of Bobo-Dioulasso, who find themselves caught between the thorny equation of land governance shared between traditional land tenure and modern land tenure. In a momentum of the city’s spatial growth, with the advance of the urban front, the population of surrounding villages can be regarded as the “losers” in this dynamic, as they endure significant pressure from the spatial expansion of the city of Bobo-Dioulasso, which involves urban actors with massive financial resources. According to Le Bris et al. (1991), the complexity of the land issue stems from the fact that land relations are social relations and therefore power relations but also permanent “negotiations” between various actors. This is evident in the interactions between indigenous populations and migrants, as well as between private economic operators and local social authorities, specifically customary chiefs. Today, this situation hinders better land governance based on customary norms which advocate for the undivided nature of the land due to speculation surrounding peri-urban real estate.

In response to this dynamic, land tenure has undergone mutations, granting a significant role to the individual at the expense of the collective regarding land ownership. Consequently, the advent of private property paves the way for the commodification of land, even though land ownership cannot be equated to the ownership of any ordinary object ( Dialla, 2003). This configuration severely tests the “social logics of the territory” ( Mathieu, 1999). In this vein, Lavigne Delville et al. (2001) argue that the question of rules and procedures for access to and control of land is decisive in areas where competition for resources within an insufficiently clear framework fosters conflict.

This article seeks to understand the changes occurring in land governance from a lineage perspective, unlike Lavigne-Delville (2002), who approached land dynamics at two levels: the local or micro-regional scale and the farm scale. This analysis addresses the dynamics of management norms by focusing on land mutations at the intra-lineage scale. It questions the dynamics of land tenure through the lens of shifting forms of intra-lineage land ownership and its subsequent effects on the social fabric, while acknowledging that there is no single “customary law” nor a closed “system”. As Ouédraogo (2024: 22) notes, even if the traditional land tenure system was defined by two main rules: the collective nature of the land and its “inalienability”, there is no static customary regime; it always evolves at varying paces depending on the context. The analysis of this research problem follows a methodological process supported by a theoretical approach to better clarify the different facets of the subject.

## Methodological approach

### Study area

The research was conducted in the villages of Nasso and Dindéresso, both located approximately 9 miles (15 kilometers) from Bobo-Dioulasso, the economic capital of Burkina Faso. These villages are considered integral parts of the city, as they are attached to District 7 (Arrondissement 7) of the Bobo-Dioulasso municipality.

The municipality features a relatively flat terrain characterized by a rocky chain, lowlands, and arable plains, with soils that are predominantly hydromorphic and highly favorable for agriculture. The climate is South-Sudanian, with relatively abundant rainfall that is unevenly distributed in both time and space. The vegetation is also South-Sudanian, consisting of wooded, tree-covered, and shrub savannas. Consequently, due to its physical framework and soil-climatic factors, the Bobo-Dioulasso municipality holds significant potential for farming and livestock activities.

In addition to environmental factors, the geographic location of the study area is an asset for human settlement and commercial activities. The municipality is located approximately 47 miles (76 kilometers) from Mali and 122 miles (197 kilometers) from the Ivory Coast by road. Data for this research were collected in the villages of Nasso and Dindéresso, as well as from administrative actors within the Bobo-Dioulasso municipality.

### Methodological framework

This research is based on a qualitative method. Qualitative research necessitates the researcher’s presence in the field. Due to its flexibility, it allows the researcher through interviews and observation to capture the practices of actors while interacting with them to understand specific aspects of social reality.

In alignment with the theoretical framework, this methodological approach facilitated the collection of data used to present and discuss the research findings. It allowed for an understanding of the positions and rationales of various actors regarding land governance within a context of shifting norms, highlighting both past and recent conceptions and practices.

### Sampling

A purposive sampling (reasoned choice) technique was selected for this study. This choice is justified by the investigation’s objectives, which aim for a significant rather than a representative sample. This approach prioritizes the depth of collected data while maintaining flexibility throughout the collection process.

Following this procedure, individuals with diverse profiles were interviewed. Qualitative interviews were ultimately conducted with:

- Customary and religious authorities.
- Indigenous farmers (including men, women, and youth).
- Livestock breeders and non-indigenous residents.
- Residents of Bobo-Dioulasso, administrative agents, and municipal authorities.
- “Key informants” selected for their specific knowledge of the study area and the research problem.

Specific criteria were defined to identify these targets: religious leadership, indigenous or non-indigenous status, age, years of residence in the locality, and status as a landowner by birth or by purchase. In total, thirty-two individual interviews were completed.

### Data collection techniques and tools

The data collection techniques and tools mobilized include:

- Semi-structured interviews using semi-structured interview guides.
- Observation using an observation grid.

As noted by Olivier de Sardan (1995), these techniques are not entirely neutral, as every form of data production has inherent advantages and disadvantages. To address this, the study employed “triangulation” of both informants and information.

The interviews focused on the central themes of the research problem. They took place in the homes of certain respondents, workplaces, administrative offices, and on farms. These tools allowed for the collection of data that clarifies the experiences and practices of actors regarding land tenure.

### Informed consent

This study was conducted among adults aged 22 and over. The sample therefore does not include minors. All interviews were conducted with the consent of the interviewees. It should be noted that consent was obtained in two forms: verbal and signed.

The verbal form is explained by the fact that the study is largely conducted in rural areas where literacy rates are high. Thus, respondents gave their consent verbally after the researcher read them the informed consent form. This form is permitted in social science data collection in rural Africa, where illiteracy is widespread. To this end, data collection from this category was carried out in strict compliance with ethical and professional standards, while ensuring confidentiality in the use and publication of the research results.

Written consent was mainly sought from educated individuals. These included civil servants in towns and villages and a few individuals who could read and write in the villages covered by the study.

In summary, the informed consent form developed as part of the study can be found in the appendices to this document.

### Ethics statement

This research was approved by the ethics committee of the Society, Mobility and Environment Laboratory (LASME) at Joseph Ki-Zerbo University in Burkina Faso. The Society, Mobility and Environment Laboratory was created in 2013 by decree n°2012-MESSRS/SG/UO/P, establishing and naming the research centers, laboratories and teams within the Doctoral School of Letters, Human Sciences and Communication (ED/LESHCO) of the University of Ouagadougou, which became Joseph Ki-Zerbo University.

This study is governed by an ethics certificate issued by the internal ethics committee of the Laboratory for Society, Mobility and Environment, whose mission is to ensure compliance with ethical and professional standards in the conduct of scientific work by laboratory members. This work may be published in journals at the national, African, and international levels for broad visibility. In this context, the research project, entitled “From Lineage Property to Individual Property: Dynamics of Land Management in the Villages of Nasso and Dindéresso, Hauts-Bassins, Burkina Faso,” received a favorable opinion from the ethics committee with the following ethics approval number: 2018-09-87388.

### Theoretical foundation

This reflection is based on Bruce’s evolutionary theory of property rights. It describes the process of rights evolving toward individualization, established following the “reduction of community control over the distribution and use of land and the increase in individual land rights of producers and right holders” ( Bruce 1986: 52). Applying the evolutionary theory of property rights to land tenure makes it possible to understand the relationships between humans and land in contexts of abundance, scarcity, and commodification.

- **In a context of abundance**: Establishing private property is not a major concern because the negative externalities caused by individual exploitation are negligible.
- **In a context of scarcity**: As the population grows and commodification intensifies, land becomes scarce, conflicts develop, and the demand for secured land rights increases.
- **The tipping point**: This process is marked by the emergence of private property rights evolving toward measures of individualization and formalization.

Addressing mutations in land tenure through the evolutionary theory of property rights requires discussing the increasingly recurring conflicts that follow scarcity, commodification, and the introduction of individual possession. This approach leads us to combine evolutionary theory with conflict-based reasoning to better understand intra-lineage land management modes and their evolution in a context of heightened competition for land appropriation.

## Results

The findings of this investigation are organized into three themes: land dynamics, the mutation of intra-community property forms, and the individualization of land leading to commodification.

### Land management through the prism of public policy

In Africa, land management is often associated with the “sacred” and is marked by legal dualism. In traditional society, land is managed by a customary tenure system centered on community social norms. This customary system is organized around traditional authorities and dominated by lineage management. It defines rules where land is considered collective heritage, with portions granted for use based on social codes. Because land rights are founded on local social values, the land issue cannot be dissociated from the established social order.

Analyzing land dynamics in Africa involves questioning the socio-political history of territories following colonial penetration. After the annexation of colonies, public policies were established by colonizers to oversee political, social, and environmental fields. New management modes were introduced to organize land according to the colonizer’s vision:

- **July 24, 1906**: A decree was adopted regarding the land registration system in Upper Volta.
- **July 26, 1932**: This decree was replaced by a new land decree that continued previous policies.

This centralized policy persisted after independence, maintaining legal provisions that often contrasted with social logics. In the Burkinabè context, a major turning point occurred with the 1983 Revolution.

Under the revolutionary regime, the Agrarian and Land Reform ^2^ (RAF) was adopted, marking a break from previous land policies. Through this mechanism, policy-makers demonstrated a will to introduce a legal framework that made the RAF an instrument for radical reorganization in land management. While the RAF was adopted to break free from the colonial yoke, it also envisioned a break from customary social norms, of which the customary chieftaincy is the primary guarantor. For the revolutionary regime, this vision aimed to liberate land from the grip of the customary chieftaincy, which was viewed as a mere usufructuary of the agrarian system. By theoretically reducing the power of customary authority, the revolution’s land policy aimed to mobilize land resources to achieve food self-sufficiency by encouraging civil servants and economic operators to return to the land.

The fall of the revolutionary regime marked a new phase in land policy reform. Consequently, the RAF was revised in accordance with the Constitution of June 11, 1991 ^3^. This new legal framework brought about an innovation by giving a primary place to the promotion of the private sector. To address the shortcomings of the 1991 RAF, a revision was adopted in 1996 ^4^. The new version of the RAF, adopted in June 2009 ^5^, provides for a rural land tenure system aimed at ensuring equitable access to rural land for all rural actors, both individuals and legal entities, while ensuring the rational and sustainable management of natural resources under State supervision, according to Article 4. In this new legal framework, unlike the 1984 RAF during the revolution which excluded the customary chieftaincy, the authority and responsibility of customary chiefs are recognized, as rural lands are divided among the State, local authorities, and individuals (Article 5). This reform offers citizens the possibility of individual land ownership.

The transition of land heritage management from the group to the individual led to the emergence of “paper” as a “property title”. Thus began an individualization of land ownership which, in the context of this study, can be explained by population growth, urban sprawl, and the increased demand for peri-urban land. This has established a climate of speculation surrounding peri-urban land in the villages of Nasso and Dindéresso due to their proximity to the city of Bobo-Dioulasso. The transition in land ownership has triggered a real craze for land on the outskirts of the city, moving from an open space to a saturated space as a result of land dynamics.

### Mutation of intra-community land ownership forms

Land tenure plays a decisive role in social changes through the processes of mutating land management rules and economic and social production systems. These mutations are determined by a set of economic, political, and social factors and actors. In this regard, there is no single “customary land law” or “system” in a closed sense, but rather socio-political regulation mechanisms regarding access to and control of the land and its resources. In the context of Nasso and Dindéresso, in addition to other forms of challenging land management norms involving a diversity of actors, a particular form of mutation of land norms is intra-lineage in nature.

### From lineage property to family property

The lineage group, regardless of the kinship system by which it is organized, is perceived as a social unit living under the authority of a leader who is generally the group’s eldest member, also known as the patriarch. Characterized by lineage solidarity surrounding a land unit, lineage-based land ownership is under the authority of the lineage head, who holds management rights shared among members according to pre-established norms ( Soro, 2009). Thus, each lineage benefits from one or several lineage land estates over which it exercises control ( Karambiri, 2022). The deep roots of land within the social, political, and economic organization led Jacob to emphasize that “men belong to the land far more than the land belongs to men” ( Jacob, 2013: 12). This interconnection is expressed among the Bobo people, according to Sawadogo (2020), through the religion of masks, which illustrates an interdependence observed within the Man-Nature-Culture triptych. However, the author continues his analysis in light of societal dynamics. He states that competition for the control of resources often leads actors to deny one another and go back on their word, as they undo and remake certain norms that previously allowed for resource governance between lineages ( Sawadogo, 2020: 140).

This dysfunction could be explained by the shift from a communal form of land management to an even more individualistic one. From this perspective, Ouédraogo (2002) highlights that while land heritage was once communal and inalienable, these principles of traditional land management now seem to be challenged by the primary actors themselves. This conception leads to a questioning of lineage land management, which Lavigne-Delville (2002) describes as a challenge to the “theory of the local,” which relies on “pragmatic rules” that are unwritten but recognized in practice. Consequently, rural actors identify with this theory, viewing these rules as “normative,” even if their actual practices do not exactly correspond to these norms and may even be quite different and much more varied ( Lavigne-Delville, 2002).

In the localities of Nasso and Dindéresso, land authority is shifting from lineage patriarchs to heads of individual families. In his research conducted four decades ago in the Yatenga region, Marchal (1985) showed that as lands reached saturation, transformations in family structures pushed for an extension of surface areas and an extensification of practices, worsening the overexploitation of space. The race for space thus paves the way for disputes and challenges to lineage management norms, viewed as too centralized, in favor of another form of management established at the family level. Consequently, there is a transition from land as lineage property to land as family property. In an interview, one farmer stated:

*“When people start selling land in a locality, it is obvious there will be fights; that is the reason for our problems. These problems have led to fights among us, the indigenous people. Often within the same large family [lineage], some want to sell the land and others do not, which is something that was never done before. There is a problem because you were born finding that your parents did not sell the land. If they had sold the land, you wouldn’t find any left for you today.”* (Interview with a resident of Dindéresso).

Following the rise of disputes within lineages, voices are being raised to demand the partitioning of lineage property. The consequence of these actions is the fragmentation of the local land. Thus, the recognition of family usage rights or family property has emerged. In response to internal quarrels, some lineages are proceeding to carve up the land among different families. This initiative can be likened to what Lavigne-Delville (2002) calls a security strategy intended to reduce conflicts between members of the same lineage. The basis for this option is that an individual cannot allow themselves to sell land that does not belong to their immediate family, provided that a distribution has been carried out based on the families that constitute the lineage. Kaboré (2008) describes the partitioning of lineage property rights as an individualization of the possession of collective spaces. This move toward individualization is explained by the fact that land authorities are constantly, or almost constantly, contested, challenged, threatened, restored, and modified. This weakens local balances, which are perpetually being reconstructed, recomposed, renamed, and re-legitimized ( Bologo, 2004). Even if this reform does not halt the monetization of land, it nonetheless acts as an obstacle preventing an individual from selling beyond the boundaries of their own property. Described as a redefinition of intra- and inter-community land rules, this reform represents an adaptive innovation to a new context.

### From family property to individual property

From lineage-based management, we are increasingly seeing a community reorganization of land that grants more freedom to individuals. This reality is not unique to our study area, as it has been widely described in the work of several researchers in different contexts. Regarding Gouin society, Nana (2018) analyzed the transition from property belonging to a group to the individual, which reflected the dynamics of land management in Gouin country. With lineage property and undivided assets, land rules being evolving norms, Lavigne-Delville (1998) states that they can undergo profound transformations. As proof, Coulibaly et al., in a context of land competition in Mali’s cotton zone, evoke the establishment of a trend toward the individualization of rights over space and resources with a regression of community forms of control ( Coulibaly et al. 2016). This analysis converges with the thesis of Soro (2009), which argues that when land becomes scarce, individuals wish to secure increasingly individualized and protected rights. This situation gives rise to disputes over rights, inheritance, and land boundaries, which in turn lead to a strong demand for more formal rights, tending toward private property.

As land is a complex issue involving the whole of society, social mutations and economic-political readjustments will impact the land tenure system due to its permeability. This leads Nana to say that land relations are defined by the overall functioning of society; they are inextricably linked to social, political, economic, ideological, and cultural relations ( Nana, 2018: 9). Consequently, the quest for land resources will increasingly pit brothers of the same lineage against each other, even though they hold the same rights to the land of their common ancestors. This could be explained by the dislocation of family units ( Ouédraogo, 2002). Thus, local land rules, which are composite and evolving in light of the social, political, and economic history of societies, will become hybridized with state rules ( Lavigne-Delville, 2002). This is a situation that could be described as the erosion of customary norms ( Benjaminsen, 2002).

Far from attributing all land reforms to modernity, it is worth noting that pre-colonial Africa knew private property, but it was carefully excluded from land relations because the emphasis was more on the collective dimension. Regarding the land domain, private land ownership is a requirement and a constraint of capitalism ( Le Roy, 1998). And so, with the dynamics of society and the erosion of social ties, land passes from a lineage asset to a family asset, then to an individual asset. This modality of ownership and acquisition establishes a new order: the individualistic management of land, supported by a taste for material goods, which has ultimately introduced new forms of relationship to the land. Hence the thought of Korbéogo (2015), who argues that cash is a vital resource in accessing land and land ownership. This new mode of land access erodes customary norms and transforms traditional logic of land exploitation ( Affessi, 2015). This new form of relationship to the land has challenged traditional conventions of soil exploitation by making land an object of sale, loan, and lease, made possible because of private property following the spread of capitalism. Indeed, the disintegration of the social fabric has allowed for a dissociation of community heritage. Thus, we are witnessing the appearance of individuals possessing personalized assets; among these assets may be common or lineage lands subject to the rule of individual appropriation, leading to their fragmentation and appropriation ( Ouédraogo, 2002). The introduction of the capitalist model, which advocates for individual exploitation and enrichment, is accompanied by an exacerbation of inter- and intra-community and even intra-family conflicts. For this young respondent from the village of Dindéresso, this situation is the source of the conflicts. He adds that:

“We have many problems today, and these problems are land problems. Now, the fighting is between family members. You will find people from the same family fighting over land. Some family members want to sell the land, and others do not agree yet. Like this, they are forced to sell because no one listens to the head of the family anymore. Everyone thinks they are free. This is the source of our problems these days.” (Interview with a young man from the village of Dindéresso).

“The individualization of land ownership is far from being a guarantee of sustainable land resource governance, let alone a way out of rural precariousness. Instead, it accentuates the impoverishment of the population, making rural residents even more precarious and dependent on city dwellers. This progressive impoverishment of rural populations can be explained by the establishment of a “differentiated logic”.

In the past, land held a central role in agricultural production and carried significant symbolic weight. Beyond these roles, it functioned as a capital stock and a reserve of assets, constituting savings for families and an investment in terms of assets. Under the new differentiated logic, these savings vanish and investments dwindle. Consequently, the new dynamics of land relations established in the study area favor the rise of land commodification, which is intensified by the race for land as urban actors enter the fray.

### When individualization opens the way to commodification

Land commodification is driven by several factors. Within the sub-regional context, improvements in production conditions, population growth, the multiplication of actors, and migration have created new dynamics in natural resource management.

Traditionally, in the African conception, land is a community good and a non-commercial collective heritage, even if it could be subject to individual appropriation. Today, it has become a commercial commodity due to the rising monetization of social relations following the erosion of social ties. In the peri-urban villages of Bobo-Dioulasso, land sales are so frequent that a nostalgia for the past, when land was not sold, has developed among the indigenous population. This rapid rise in land monetization reduces the area of cultivable land and establishes land insecurity.

This climate of land insecurity intensifies following land acquisitions by new actors, a situation that exacerbates competition for land and increases its market value. This environment, where land sales are permitted, encourages the disappearance of family land heritage as new actors arrive.

### The shift from inalienability to market speculation

Sales that were forbidden yesterday due to the inalienable nature of land are undergoing a profound mutation today in the villages of Nasso and Dindéresso. In many families, land commodification is becoming increasingly widespread, treating land as a commodity just like any other. This reality supports the analysis that the idea of “land as community heritage that cannot be sold” is a primary, communitarian conception that views land as mere matter rather than a resource.

As a resource available to the highest bidder, land is subject to speculation that disintegrates the social fabric. Interviewed actors have compared this situation to a “time bomb” because land monetization has become a trend. It fosters a conflictual climate, with numerous recorded disputes between individuals of the same family in Nasso and Dindéresso following land sales. These conflicts frequently jeopardize the balance of the entire community. The testimonies of the surveyed individuals illustrate the establishment of land commodification as a primary mode of land access.

“In the past, land was not sold, but nowadays, money and the desire for it drive people to start selling their land. Some sell it to buy a motorcycle or a car. I want a motorcycle, and I want a car too, but I will never sell the land because I was born into this land and my parents did not sell it, so I am not going to sell it.” (Interview with a community leader in Dindéresso).

“These days, people have become autonomous and land sales have increased significantly. Selling land has become like a trend for people. The reason for selling land is the craving for money. It is difficult for some to resist when they hear about money. Now the selling price has gone up; otherwise, in the past, a hectare was sold at ridiculous prices, between 50,000 and 100,000 francs in the 2000s. But nowadays, if you don’t have 300,000 or even 500,000 francs, you cannot get a hectare because there is no land left. If you lack the means and find yourself in a difficult situation, and a wealthy person makes you an offer, you will sell your land. That is the real problem currently in our village. Even tomorrow, people will do it, even though they know that land should not be sold.” (Interview with a farmer in Nasso).

According to the excerpts from the interviews, land speculation benefits the wealthiest classes. As a woman in Dindéresso puts it, “the rich come from the city to flatter the poor and buy their land.” The argument of “flattery” does not infantilize the locals; rather, it takes its meaning from the sometimes-derisory amounts received in exchange for transferring usage and property rights to a third party. With the erosion of social ties and quarrels between family members, land is shifting from a family asset to an individual one, making its commercialization possible. This reality establishes a new order: the individualistic management of natural resources. According to Lavigne-Delville (2002), the craze for land involves internal social relations within local rural society, but also the relations between the State and its citizens and, increasingly, between rural residents and urban actors with land interests in rural areas. Consequently, land appears as an arena that brings actors and their logic of land governance and appropriation into confrontation.

## Discussion

Analysis of the research results highlights a central question that requires elucidation: the challenge to the sacred nature of land, which serves as a principle of land security according to the philosophy of African communities. This logic is rooted in the theory of land sacrality, viewing land as a collective and inalienable good. The significance accorded to this resource made it a fundamental entity in traditional social organization. Natural resources possess mythological, sacred, and religious dimensions, leading to the dedication of cults and rites to them because they are believed to possess a soul. This supports the idea from Dévérin (1998) that among the Mossi of Burkina Faso, land is simultaneously the property of humans and spirits. This reality is shared by several African communities and even extends beyond the African continent. Research by Adou (2015) on the Kyaman, an indigenous ethnic group in Abidjan (Ivory Coast), shows that land is the foundation of society and the creative source of life. To this end, it is governed by supernatural powers to allow it to bring forth and shelter the other elements of the universe. Far from being a purely Afrocentric perspective, Tola (2014) notes from the other side of the Atlantic that the expression “land is our life” is the slogan repeated by the overwhelming majority of Amerindian peoples.

This “double ownership” reinforces the sacred character of the land while imposing strictness in its management to ensure social harmony. Relationships to land are experienced as an essential aspect of the social and cultural identity of groups and individuals; this link creates an attachment to the territory that is inseparable from community belonging. Similarly, Nana (2018) asserts that land involves intangible aspects of social life, such as power, the identity of groups and individuals, and the relationship to the sacred. In light of these analyses and referring to Bobo society, we can affirm that land is a social, political, cultural, economic, and religious cornerstone in the life of the community. However, with the erosion of social ties, land is progressively losing its status as a communal good to become an individual good, a mutation that makes its commercialization possible.

The shift from a collective to an individual good occurred due to changes in land ownership forms at the community and lineage levels. Today, a new dynamic in land relations is underway, linked to heavy pressure on land, which increasingly limits access modes based on traditional land customs. Although Chauveau (2006) maintains that land has a predominant social significance in the customary vision, lands today are rarely publicly discussed as commodities, even though they are subject to bargaining with costs set according to several criteria. This situation arises from what can be called the demystification of customary norms governing land.

The desacralization of land can be analyzed through Bruce’s evolutionary theory of property rights, as changes in land access forms and modes lead to land being perceived as a commodity. For Bruce (1986), the evolution of land rights reduces community control and distribution of land. In this sense, the transition from “sacred land” (an inalienable resource) to “object land” (a marketable good) has caused social upheaval and cognitive shifts regarding land tenure in the villages of Nasso and Dindéresso. Consequently, one can now speak of an “emerging” land market that represents a rupture with customary land principles.

However, according to Zongo (2009), land sales constitute a source of land insecurity and conflict because these sales often occur in an “informal market” characterized by a lack of clarity and transparency. For Bonnet-Bontemps (2003), addressing land commodification involves discussing factors such as demographic pressure and the increasing commercialization of land products. Faced with this reality, there is increased competition and pressure from a growing number of users for the use or appropriation of land resources, resulting in the weakening of the legitimacy and power of traditional land authorities in many villages.

Land security remains indispensable for social peace. While the emphasis on land conflicts should not lead to an idealization of a past where an “ideal community” lived in perfect harmony, the mutation of land tenure has significantly impacted the social fabric and generated numerous conflicts in the study area. Thus, conflict appears as a revelator of societal dynamism and a major factor in the control of land resources. In a context of shrinking land reserves and a desire for wealth, Kaboré (2020) states that some indigenous people, having exhausted their own land reserves, deceive buyers by ceding land they do not fully own because they share it with other rights-holders who have no desire to sell.

## Conclusion

At the conclusion of this analysis, it is clear that land is a cross-cutting issue that impacts all spheres of society. Consequently, it involves multiple and multifaceted stakes, particularly in the fields of governance, the security of movable and immovable property, and social cohesion. To this end, land tenure security is indispensable for the construction and consolidation of social cohesion between both intra-community and extra-community members. This justifies the place that land occupies in social organization and the role played by the guardians of land governance in traditional society. In this framework, land was considered a collective heritage, an undivided asset under the authority of the land chief and patriarchs.

Following the arrival of colonizers in the 19th century, social structures underwent numerous mutations, as the colonial political vision challenged the modes of structuring and governance of former colonies through the formalization of French West Africa. However, this policy did not emerge in a vacuum devoid of governance norms; this led to the establishment of legal dualism in land management within the collective memory, which plunges the citizen into two sets of legal norms: traditional and modern land management. Given its importance and interest, mutations in land tenure have triggered social changes through processes that challenge land management rules and socio-economic production systems.

In the face of this dynamic, land tenure has undergone mutations that grant a significant place to the individual at the expense of the collective regarding land ownership. We are thus witnessing the advent of private property, which paves the way for the commodification of land, turning it into a marketable good. This concept has introduced a significant change in social organization and in the relationship between people and the customary rules and norms that govern land. In the villages of Nasso and Dindéresso, alongside other forms of challenging land management norms involving a diversity of actors, a specific form of mutation in land norms is occurring at the intra-lineage level.

From lineage-based management, we observe a reorganization of community land management that grants more freedom to individuals, leading to the transition from lineage property to family property, and subsequently to individual property. This situation has been made possible by the fragmentation of property rights, which allows for greater individualization of the possession of collective lands. While land in the African conception was considered a community asset and a non-commercial collective heritage, today it has become a commercial good due to the rise of the monetization of social relations following the erosion of social ties. This new problem plunges communities, especially rural ones, into disarray and foreshadows the emergence of a society of peasants without cultivable land.

## Ethics declaration

As part of this research, we collected data from stakeholders defined in accordance with the study’s objectives. Interviews were conducted with various actors in the study areas. We obtained authorization and approval from the ethics committee of the Laboratory for Society, Mobility and Environment (LASME) at Joseph Ki-Zerbo University to conduct surveys with the target populations. The authorization and ethics certificate are included in the documents accessible to publishers and readers via the document submission link.

In accordance with ethical requirements and the need to respect the anonymity of interviewees, the research ethics committee (REC) requires the researcher to strictly adhere to the following principles:

- The names and surnames of the interviewees will not be published, but their profiles can be published anonymously;
- Audio recordings and full transcripts of interviews may not be disseminated, but the data used to prepare a scientific article may be made available to publishers and readers. This includes tables of variables, profiles of interviewees, excerpts from transcripts, and documents that attest to the study’s compliance. Publishers, reviewers, and readers can access these documents via the document submission link.

## Declaration of consent

This study was conducted in the form of a survey of various stakeholders. To this end, an informed consent form was drawn up in order to obtain the approval of the various respondents before the start of each interview. The consent form can be found among the documents available for consultation via the c-dissous link.

## Data Availability

This study is based on primary and secondary data. The primary data comes from interviews conducted with the stakeholders defined in the methodology. The secondary data consists of a literature review, the references for which are listed in the bibliography. As for the primary data, for ethical reasons and due to commitments made to the respondents, the transcribed interviews cannot be submitted in their entirety, but the analysis list highlighting the variables and verbatim extracts will be made available to publishers and readers. All sources and documents relevant to this study are available for consultation. These items are provided as supplementary data (appendix). Reference: manually annotated corpus From Lineage Property to Individual Property: Dynamics of Land Management in the Villages of Nasso and Dindéresso, Hauts-Bassins, Burkina Faso https://doi.org/10.5281/zenodo.18647915. This project contains the following underlying data: Data file 1. (Description of data.) Data file 2. (Description of data.) The data is available under the terms of the Creative Commons Attribution 4.0 International (CC-BY 4.0) license.

## Footnotes

^1^
“Established by a decree on June 16, 1895, under the direction of a governor-general, French West Africa (A-OF) addressed the need to coordinate French penetration into the interior of the African continent under a single authority.”

^2^
The RAF was adopted through Ordinance No. 84-050/CNR/PRES on August 4, 1984.

^3^
Decree No. AN VIII-0328 TER/FP/PRES/PLAN/Coop of June 4, 1991, regarding the implementation of the Agrarian and Land Reform (RAF) in Burkina Faso.

^4^
Law No. 020/96/ADP of July 10, 1996, establishing a land-use tax for the occupation and enjoyment of lands within the national land domain belonging to the State.

^5^
Law No. 034-2009/AN of June 16, 2009, regarding the rural land tenure system in Burkina Faso.
