# Supplementary material for: From Lineage Property to Individual Property: Dynamics of Land Management in the Villages of Nasso and Dindéresso, Hauts-Bassins, Burkina Faso
Source: Open Res Afr. 2026 Mar 2;9:8. [Version 1] doi: 10.12688/openresafrica.16420.1 (PMC13032100; doi:10.12688/openresafrica.16420.1)
Supplement: Peer review history

# Reviewer report: Reviewer response for version 1

**DOI:** 10.21956/openresafrica.17605.r33986

**Published:** 2026-04-01

**Authors:** Malcolm Childress

**Affiliations:** 1 Global Land Alliance, Washington, USA

This is a well-written, valuable contribution to the discourse on the evolution of traditional land rights towards individualization and commodification of land rights in the context of peri-urban expansion in Burkina Faso. It is well-grounded in the literature on the evolution of land rights systems, and does an solid job of explaining the context of traditional lineage systems evolving toward individualization as new financial incentives come into play. A few points of critique: 1) the methods section does not give details on the informants who were sampled. In a small (n=32) purposive sample for a qualitative study, it would be helpful to know how many of each category/profile were interviewed and more about their characteristics (for example, age and sex) and it would help to understand how the themes were generated (for example by coding or textual analysis of transcripts); 2) the qualitative results from the primary interviews are very sparsely used in the text, with only a couple quotations, yet these are the most powerful and convincing elements in the manuscript. It would be helpful for each of the main points to be supported by verbatim quotes; 3) while the thrust on the overall  process of movement-- from a lineage system to mixed system with much greater prevalence of individualization is strong throughout-- the manuscript gives very little treatment to the actual internal lineage and family dynamics that drive the process, with little account of how outcomes are reached (for example, gender and age dynamics, intra-household bargaining, locational and price considerations); 4) there is almost no treatment of the policy context and how either traditional or statutory authorities may or may not be attempting to intervene in the processes described. Overall this is a valuable contribution that would benefit from small revisions in these areas to become more grounded in its own empirical data and to shed further light on the family-level and market-level social dynamics which it is examining.

Is the work clearly and accurately presented and does it cite the current literature?

Yes

If applicable, is the statistical analysis and its interpretation appropriate?

Not applicable

Are all the source data underlying the results available to ensure full reproducibility?

Partly

Is the study design appropriate and is the work technically sound?

Yes

Are the conclusions drawn adequately supported by the results?

Yes

Are sufficient details of methods and analysis provided to allow replication by others?

Partly

Reviewer Expertise:

Land governance and land policy, land market analysis, land information systems, property taxation, spatial planning.

I confirm that I have read this submission and believe that I have an appropriate level of expertise to confirm that it is of an acceptable scientific standard, however I have significant reservations, as outlined above.

# Reviewer report: Reviewer response for version 1

**DOI:** 10.21956/openresafrica.17605.r33982

**Published:** 2026-03-30

**Authors:** Kehinde Hassan Babalola

**Affiliations:** 1 Cape Peninsula University of Technology, Cape Town, South Africa

**Introduction**

 First, the introduction must be revised in depth to present the subject of the paper, as well as the questions supporting the research carried out. There is a need for an introduction that provides more background for the study. There are several terms used without explaining their meaning to the reader. Several of the references used in the Introduction are very old. The research aim and objectives are not very clear to the reader. I suggest you make it clear by stating the research aim is....... What are the research questions that the study is addressing?

**Methodological approach**

 The presentation of the methodology also raises some confusion, since the subject of the paper is not clearly presented in the introduction. The case study method is well presented, but in a very general way. There is a need to detail the Bobo-Dioulasso case study, explaining why it is a justified choice for the study of land management using the mentioned evolutionary theory. Also, using the case study method, it would be necessary to add a section presenting the characteristics of Bobo-Dioulasso, the legislative framework and the institutional organisation of land administration. The interviews with representatives of various organisations and with land rights holders must be detailed from a methodological point of view.

 The study area description needs to be shown on a map for readers who are not familiar with this area. What do you mean by climate and vegetation are South-Sudanian? How the sampling technique was applied is not explained to the reader. Data collection modes were not explained. How was the data collected, and with what? Why use verbal consent when most respondents are learned? How was the collected data analysed? How do you determine the reliability and validity of this research? Overall, the methodology is not scientifically sound and cannot be replicated.

**Discussion**

 The problem mentioned is not unique to Burkina Faso; the study will benefit from a more comprehensive evaluation of the issue. This will enhance the conversation even more by making it possible to relate Burkina Faso's solution to the rest of the world and the scientific community. Because the issue has been concentrated on Burkina Faso and appears to be more of a societal issue than a research issue, the knowledge gap has not been thoroughly identified. The results have also not been discussed due to the study's minimal relevance to the scientific community. As a result, the study's findings are entirely restricted to Burkina Faso, and its scientific value is not demonstrated. The study's results are discussed by the author or authors in relation to the field's state of the art. Although some articles are cited in the discussion section, the authors do not explicitly state if these studies relate to Burkina Faso. I will suggest a robust discussion where similar issues identified in SSA and the Global North are discussed.

**Conclusion**

 The conclusion section must not only highlight the findings but must also direct policy implications of these findings

Is the work clearly and accurately presented and does it cite the current literature?

Partly

If applicable, is the statistical analysis and its interpretation appropriate?

Not applicable

Are all the source data underlying the results available to ensure full reproducibility?

Partly

Is the study design appropriate and is the work technically sound?

Partly

Are the conclusions drawn adequately supported by the results?

Partly

Are sufficient details of methods and analysis provided to allow replication by others?

Partly

Reviewer Expertise:

NA

I confirm that I have read this submission and believe that I have an appropriate level of expertise to confirm that it is of an acceptable scientific standard, however I have significant reservations, as outlined above.

# Reviewer report: Reviewer response for version 1

**DOI:** 10.21956/openresafrica.17605.r33984

**Published:** 2026-03-28

**Authors:** Sukmo Pinuji

**Affiliations:** 1 Technical University of Munich, Munich, Germany; 2 Sekolah Tinggi Pertanahan Nasional, Yogyakarta, Special Region of Yogyakarta, Indonesia

This manuscript addresses interesting topic on land tenure transformation under peri-urban pressures in Burkina Faso. However, several aspects need to be strenghtened to improve its academic rigor and contribution. 

 1. Novelty and contribution. 

 The paper remains largely descriptive. The novelty and specific contribution are not clearly articulated, particularly in relation to existing literature and broader international land management debates. This should be made explicit. 

 2. Linking national context and case study. 

 The connection between national land dynamics and the village-level analysis (Nasso and Dinderesso) is unclear. The manuscript would benefit from clarifying this linkage, as well as providing clear rationale for selecting these case study sites. 

 3. Methodological clarity. 

 The metods section lacks of sufficient detail to ensure transparency and reproducibility. Please clarify: (1) sampling strategy and respondent selection criteria in more detailed way ; (2) number and profile of respondents; (3) data analysis procedures; (4) how triangluation was conducted

 4. Operationalization of the theoy 

 While property rights theory is mentioned, its application in the analysis is limited. The manuscript shoudl better demonstrate how theoretical concepts inform the empirical analysis. This should also applies in method section. 

 5. A diagram illustrating the evolution of land management systems (e.g., lineage --> family --> individual ownership) would improve clarity and help structure the findings. 

 6. Given the diversity of respondents, the manuscript should address potential biases and differing perspectives, and reflect these dynamics more explicitly in the analysis/ discussion. 

 7. The manuscript would benefit from a clearer articulation of policy implications. While the findings highlight the shift toward individualized and commodified land systems, it is remain unclear how these insight inform land governance reforms/ management of peri-urban land pressures/ balancing customary and statutory system. Explicit, actionable policy recommendations would enhance the paper's practical relevance. 

 In conclusion, the paper has potential but requires substantial revision to strenghten its conceptual clarity, methodological rigor, analytical depth, and policy relevance.

Is the work clearly and accurately presented and does it cite the current literature?

Yes

If applicable, is the statistical analysis and its interpretation appropriate?

Not applicable

Are all the source data underlying the results available to ensure full reproducibility?

Partly

Is the study design appropriate and is the work technically sound?

Yes

Are the conclusions drawn adequately supported by the results?

Yes

Are sufficient details of methods and analysis provided to allow replication by others?

Partly

Reviewer Expertise:

land management, land acquisition, livelihood restoration, land governance

I confirm that I have read this submission and believe that I have an appropriate level of expertise to confirm that it is of an acceptable scientific standard, however I have significant reservations, as outlined above.

# Reviewer report: Reviewer response for version 1

**DOI:** 10.21956/openresafrica.17605.r33910

**Published:** 2026-03-27

**Authors:** Raogo Noël Gansaonre

**Affiliations:** 1 Nazi Boni University, Bobo-Dioulasso, Burkina Faso

**Abstract**

 In the results section, specify the consequences of the privatisation of land ownership.

**Study area**

 Include a location map. In the description of the study area, the author does not emphasise the dynamics that contribute to increasing land pressure (population growth, changes in agro-pastoral activities, urban development, etc.).

**Data collection**

 Data collection was carried out in two villages and involved 32 people. The author fails to specify the number of people per village and per category.

**Results**

 The article discusses changes in land tenure systems brought about by land pressure in the peri-urban areas of the city of Bobo-Dioulasso in Burkina Faso. The author shows that tenure forms have shifted from collective management to individual appropriation, which opens the door to land sales and subsequently to land speculation. The article provides very interesting insights into the dynamics at play in peri-urban areas in Burkina Faso.

 However, the author remains rather silent on the processes leading to these various changes. Specifically, how does one move from collective to individual ownership?

 The author does not really address the factors that explain the sale of land. 

 The author makes little use of survey data and focuses on the literature.

Is the work clearly and accurately presented and does it cite the current literature?

Yes

If applicable, is the statistical analysis and its interpretation appropriate?

Not applicable

Are all the source data underlying the results available to ensure full reproducibility?

Yes

Is the study design appropriate and is the work technically sound?

Yes

Are the conclusions drawn adequately supported by the results?

Yes

Are sufficient details of methods and analysis provided to allow replication by others?

Yes

Reviewer Expertise:

All of comments are minor and can be take account by the author

I confirm that I have read this submission and believe that I have an appropriate level of expertise to confirm that it is of an acceptable scientific standard.

# Reviewer report: Reviewer response for version 1

**DOI:** 10.21956/openresafrica.17605.r33906

**Published:** 2026-03-17

**Authors:** Yacouba Tengueri

**Affiliations:** 1 Sociology and anthropology, Daniel Ouezzin Coulibaly University, Dedougou, Dédugou/Bankuy, Burkina Faso

**Methodology**

**1. Insufficient justification for site Selection**

 The villages of Nasso and Dindéresso were not selected based on explicit criteria related to land tenure issues. The reader is left wondering why these two sites were chosen rather than other areas on the outskirts of Bobo-Dioulasso. Add a map to locate the study area, allowing a foreign reader to better understand the geographical situation.

**2. Overly generic qualitative framework**

 The precise type of qualitative approach used (case study, ethnography, grounded theory, etc.) is not identified, making the methodology difficult to evaluate and unlikely to be replicated.

**3. Lack of theoretical saturation**

 The 32 interviews are not justified by any recognized methodological criteria. The concept of theoretical saturation, standard in qualitative research, is entirely absent.

**4. Opaque sample distribution**

 The distribution of interviews among the different categories of stakeholders is not specified, making it impossible to assess any potential imbalance in representation.

**5. Operationalization of selection criteria unexplained**

 The criteria for identifying respondents are listed, but their concrete implementation in the field is not described.

**6. Gender absent as an analytical variable**

 Despite the well-documented gender inequalities in access to land in West Africa, gender is not treated as a structuring variable in the sampling.

**7. Researcher reflexivity lacked**

 The researcher's stance, background, and conditions of access to the field, fundamental elements in qualitative research, are not discussed.

 The type of interviews, the existence of interview guides, and the methods of recording, transcribing, and translating the data are not specified.

**8. Bibliography**

 Lack of references for underlying data and extended data

Is the work clearly and accurately presented and does it cite the current literature?

Yes

If applicable, is the statistical analysis and its interpretation appropriate?

Not applicable

Are all the source data underlying the results available to ensure full reproducibility?

Yes

Is the study design appropriate and is the work technically sound?

Yes

Are the conclusions drawn adequately supported by the results?

Yes

Are sufficient details of methods and analysis provided to allow replication by others?

Partly

Reviewer Expertise:

Gender and development, land, forced displacement

I confirm that I have read this submission and believe that I have an appropriate level of expertise to confirm that it is of an acceptable scientific standard.
